# Supplementation of *Alternanthera sissoo* pellets on feed digestion, rumen fermentation, and protozoal population in Thai native beef cattle

**DOI:** 10.1016/j.heliyon.2024.e29972

**Published:** 2024-04-20

**Authors:** Sukruthai Sommai, Metha Wanapat, Chanon Suntara, Rittikeard Prachumchai, Anusorn Cherdthong

**Affiliations:** a Tropical Feed Resources Research and Development Center (TROFREC), Department of Animal Science, Faculty of Agriculture, Khon Kaen University, Khon Kaen, 40002, Thailand; b Department of Animal Science, Faculty of Agricultural Technology, Rajamangala, University of Technology Thanyaburi, Pathum Thani, 12130, Thailand

**Keywords:** Brazilian spinach, Digestibility, Flavonoid, Rumen microorganism, Ruminant

## Abstract

The objective of this experiment was to study the effects of Brazilian spinach (*Alternanthera sissoo*) pellet (BSP) supplementation on rumen fermentation, protozoal population, and methane (CH_4_) estimation in beef cattle. Four male Thai native beef cattle, 3 years old, with an average bodyweight of 180 ± 5 kg, were randomly arranged in a 4 × 4 Latin square design. The cattle were supplemented (on-top) with four levels of BSP (2, 4, 6, and 8% dry matter intake (DMI), respectively). The roughage component, derived from rice straw, was fed at 40 % of DMI, while the concentrate diet was fed at 60 % of DMI. The result of the experiment demonstrated that BSP supplementation had no effect on the DMI, nutrient intake, or nutrient digestibility (p > 0.05). Rumen pH and ammonia-nitrogen concentration were not significant, while the average protozoal population linearly decreased (p = 0.002) with BSP supplementation. Mean blood urea-nitrogen concentration was linearly increased (p = 0.004) when increasing the level of BSP. Brazilian spinach pellet had no significant effect on total volatile fatty acids (TVFA), VFA profiles, and CH_4_ estimation (p > 0.05). Nitrogen balance was no different from the supplementation of BSP. The study indicates that Brazilian spinach pellet supplementation showed no noticeable effects on feed intake, rumen parameters, and nitrogen utilization; however, at 6–8% of DMI, there was a decrease in protozoal population, with no corresponding reduction in CH_4_ estimation.

## 1 Introduction

A diverse group of plant polyphenols with a wide range of biological and pharmacological properties includes flavonoids, which are found in many different plant species. The majority of plant species that are utilized as animal feed are abundant sources of flavonoids and other polyphenolic compounds [1]. Hassan et al. [2] revealed that flavonoids influence rumen microbiology, fermentation, and metabolic status to improve ruminant health and productivity. Chen et al. [3] demonstrated that plant containing of flavonoids could increase the utilization and digestion of nutrients as well as energy. Flavonoids have been suggested as an alternative to antimicrobial treatments to obviate rumen acidosis in beef on high-concentrate diets [4]. As per Balcells et al. [5], young steers received a concentrated Bioflavex® mixture derived from citrus extracts, specifically flavonoids sourced from Citrus aurantium and Citrus paradisi. This regimen positively influenced the rumen ecology, preventing decreases in ruminal pH, increasing propionate levels, and decreasing acetate concentrations. The observed flavonoid effects in the rumen could be attributed in part to the augmentation of lactate-absorbing bacteria [6]. According to Oskoueian et al. [7], it was observed that flavonoids led to a reduction in the *in vitro* release of methane (CH_4_) and ciliate protozoa. Rumen protozoa serve as hosts for methanogens, facilitating the occurrence of methanogenesis, the biochemical process responsible for methane production [4]. By decreasing the number of rumen protozoa, which function as hosts for methanogen bacteria, there may be a simultaneous reduction in methanogenesis [5,7].

Brazilian spinach (BS) is thought to be a terrific supplement with great feed effectiveness. According to Sommai et al. [8], in order to achieve a high total flavonoid content, BS was harvested between fifteen and thirty days following planting and regrowth. Their findings indicated that flavonoids extracted from BS supplementation led to elevated contents of TVFA and propionic acid (C3), while simultaneously reducing CH_4_ levels as well as population of protozoa. Nevertheless, it is necessary to conduct an in vivo investigation to validate the outcome of this discovery. Additionally, there is insufficient data regarding the incorporation of BS into various feeding regimens as a feed pellet. The hypothesis was that BSP may enhance efficiency of ruminal fermentation, lowering the number of protozoa and minimize CH_4_ synthesis. The present experiment aims to find out how BSP supplementation affects rumen fermentation, protozoal population, and CH_4_ estimation in Thai native beef cattle.

## 2 Materials and methods

### 2.1 Animal care and experiment location

All trials were carried out in accordance with the National Research Council of Thailand's Ethics of Animal Experimentation and the Committee of Animal Experimentation on Animal Welfare for Experimental Animals of Khon Kaen University (IACUC-KKU-43/63). The management of the cattle was entrusted to the committee.

### 2.2 Making of Brazilian spinach pellets and total flavonoid analysis

A sample of recently harvested Brazilian spinach (BS) was obtained from TROFREC, KKU, Thailand. The accumulated biomass of BS (including leaves and stalks) was subsequently subjected to solar exposure for a period of 2–3 days, resulting in the desiccation of the material to a minimum dry matter content of 94%. In order to incorporate it into the pellet, the dry BS was ground into a fine powder using a Cyclotech Mill with a 1-mm screen size (Cyclotech Mill, Tecator, Hoganas, Sweden). The BSP sample consisted of 100 kg DM of ground BS material with a water content of 25.32%. The pelleting process was carried out using a Ryuzoukun small machine manufactured by Kakiuchi Co., Ltd. in Nakajima, Japan. After undergoing a drying process of 2–3 days, the BSP was thereafter placed in sizable plastic containers for the purpose of feeding. Analysis of the total flavonoid content (TFC) was conducted using the methodology described by Sommai et al. (2021).

### 2.3 Animals, diets, and experimental design

Four male cattle, each 3 years old with a BW of 180 ± 5 kg, were randomly assigned according to a 4 × 4 Latin square design. The cattle were supplemented (on-top) with four levels of Brazilian spinach pellets (2, 4, 6, and 8% DMI, respectively). To mitigate this risk and ensure the accuracy of supplementation levels, rigorous measures were implemented to monitor feed intake and account for any remaining feed residues, commonly referred to as orts. The absence of remnants in this study indicates that animals consumed the entire provided feed, minimizing the likelihood of an inaccurate assessment of BSP intake. Prior to initiating the experiment, training sessions were conducted with the animals to evaluate their DMI and precisely quantify their food consumption, ensuring it fell within the prescribed range of 2–8%. Additionally, to facilitate animals in ingesting a balanced proportion of BSP within the 2–8% range, initial BSP supplementation was provided, followed by subsequent feeding of roughage and concentrate once BSP levels were depleted.

The roughage component (R), derived from rice straw, was fed at 40% of DMI, while the concentrate diet (C) was fed at 60% of DMI. The cattle were put in their own pen and given free access to clean water and mineral blocks. They were fed twice a day, at 7:00 a.m. and 16:00 p.m. During the course of the experiment, there were four distinct periods: the first fourteen days were dedicated to the process of treatment adaption, and the final seven days of each period were devoted to the evaluation of nutrient digestibility and the collection of samples. Table 1 provides an overview of the chemical compositions and components of both the diets and the BSP. Following the recommendations made by the Working Committee of Thai Feeding Standards for Ruminants (WTSR), the concentrated diet was developed for the purpose of providing Thai-native beef cattle with the nutrients they require. In the current study, the concentrate diet was intended to have a CP of 154.6 g/kg DM. The CP, NDF, ADF, and total flavonoid content in BSP contain 177.4, 401.3, 327.2, and 84.2 g/kg DM, respectively.

**Table 1:** Ingredients and chemical composition of concentrate, rice straw and Brazilian spinach pellet (BSP) used for Thai native beef cattle experiment.

Item	Concentrate diet	Rice straw	BSP
Ingredients, g/kg DM			
Cassava chip	530	**-**	**-**
Soybean meal	165	**-**	**-**
Rice bran	120	**-**	**-**
Palm kernel meal	136	**-**	**-**
Urea	10	**-**	**-**
Premix^a^	10	**-**	**-**
Molasses	14	**-**	**-**
Sulfur	5	**-**	**-**
Salt	10	**-**	**-**
Chemical composition			
Dry matter, g/kg	928	926	908
Organic matter, g/kg DM	959	905.1	982
Crude protein, g/kg DM	140.6	29.0	177.4
Neutral detergent fiber, g/kg DM	343.5	716	401.3
Acid detergent fiber, g/kg DM	288.5	558	327.2
Total flavonoid content, g/kg DM	–	–	84.2

a Mineral and vitamins (each kg contain): vitamin A = 10,0000,000 IU; vitamin E = 70,000 IU; vitamin D = 1,600,000 IU; Fe = 50 g; Zn = 40 g; Mn = 40 g; Co = 0.1 g; Cu = 10 g; Se = 0.1 g; I = 0.5 g; DM = Dry matter.

### 2.4 Data collection and sampling procedures

Every day, the amount of feed that each cattle consumed was recorded by weighing the amount of feed that was supplied to them and the amount that they refused to consume. During the course of the trial, there were four phases, each lasting for 21 days, and there were also 14 days set up for preparatory adaptation. Finally, the animals were moved to the metabolism crate to ensure that entire digestion data could be collected from their urine and feces during the course of the final seven days of the period. This was accomplished through the utilization of a total collection approach. At a rate of five percent of the total fresh weight, the feces samples were collected and then divided into two parts; the first part was utilized for the examination of the DM. During the process of analyzing the chemical composition and determining the digestibility of nutrients, the second part was frozen and then blended according to animal and time. To find out what chemicals were in the frozen samples, they were defrosted, dried in an oven at 60 °C, and then ground up through a 1 mm sieve using the AOAC [9] method. The chemicals that were found were dry matter (DM; ID 967.03), ash (ID 492.05), crude protein (CP; ID 984.13), acid detergent fiber (ADF), and neutral detergent fiber (NDF), as described by Van Soest et al. [10]. The urine samples were put into a container with about 500 ml of a 10% H_2_SO_4_ solution. The container was then put in the freezer at −20°C so that the urine nitrogen could be analyzed using the Kjeldahl method according to AOAC [11] to find out how nitrogen was used. The nitrogen balance was calculated according to Ribeiro et al. [12] as follows:

(1) N -absorption (g) = N intake (g) – N in feces (g)

(2) N -retained (g) = N intake (g) – N in feces (g) – N in urine (g)

On the final day of each period, 10 mL of blood samples were collected from the jugular vein, and samples of rumen fluid were taken before feeding and at 4 h after feeding. For the purpose of assessing blood urea nitrogen (BUN) in accordance with Crocker [13], the blood samples were kept in ethylenediaminetetraacetic acid (EDTA) as an anticoagulant. The plasma that was obtained was then centrifuged at a speed of 500 g per minute for 10 min at a temperature of 4 °C. For the purpose of collecting rumen fluid samples, a stomach tube that was connected to a suction pump was utilized to collect 200 mL of fluid. A HANNA pH meter (HANNA, HI 8424, Hanna Instruments, Kallang Way, Singapore) was utilized in order to conduct an instantaneous estimation of the ruminal pH. The rumen fluid samples were put through four layers of cheesecloth and then split in twoThe initial portion, consisting of 45 mL of rumen fluid samples, was combined with 5 mL of a 0.1 M H_2_SO_4_ solution at a ratio of 9:1. After centrifuging the sample solution at 16,000×*g* for 15 min, the supernatant was collected and stored at a temperature of −20 °C. This was done in preparation for the subsequent use of the micro-Kjeldahl method for NH_3_ analysis [11]. Additionally, the concentrations of VFAs were determined by employing gas chromatography (GC; Shimadzu, Model: GC-2014, Kyoto, Japan). Particularly, the concentrations of acetic acid (C2), propionic acid (C3), and butyric acid (C4) were determined. According to Moss et al. [14], the equation that was used to determine the amount of methane (CH_4_) concentration was as follows:

(3) CH_4_ (mmol/L) = 0.45 (C2, mmol/L) - 0.275 (C3, mmol/L) + 0.4 (C4, mmol/L)

In the second stage of the experiment, 1 mL of rumen fluid was combined with 9 mL of a 10% formalin solution at a ratio of 1:9. This mixture was used to determine the protozoal population using a hemocytometer (Boeco, Hamburng, Germany) [15].

### 2.5 Statistical analysis

All data were statistically analyzed as a 4 × 4 Latin square using the General Linear Model (GLM) procedures of SAS Statistical Analysis Systems (SAS) [16]. The data was analyzed using the model:

(4) *Y*_*ijk*_ = *μ* + *M*_*i*_*+A*_*j*_*+P*_*k*_ + *ɛ*_*ijk*_

Where Y_ijk_, observation from animal j, receiving diet i, in period k, μ, the overall mean, Mi, the effect of the levels of flavonoid supplementation (i = 2, 4, 6 and 8% of DM diet), A_j_, the effect of the animal (j = 1, 2, 3, 4), P_k_, the effect of the period (k = 1, 2, 3, 4), and ɛ_ijk_ the residual effect. The results are shown as mean values with the standard error of the mean. The orthogonal polynomial contrasts were conducted to evaluate the effect of BSP supplementation doses, and differences among means with P < 0.05 were represented as statistically significant differences.

## 3 Results

### 3.1 Dry matter intake and nutrient digestibility

Adding BSP to beef cattle had no effect on nutrient intake or nutrient digestibility (p > 0.05; Table 2). DMI averaged from 5.62 to 6.08 kg/day and 2.42–2.62% of BW (p > 0.05).

**Table 2:** Effect of Brazilian spinach pellet supplementation on feed intake and digestibility of nutrients in beef cattle.

Items	BSP supplementation (%) of DMI				SEM	Contrast		
	2	4	6	8		L	Q	C
Total DM intake								
kg/day	6.08	6.05	6.03	5.62	0.30	0.31	0.53	0.75
% of BW	2.62	2.61	2.55	2.42	0.14	0.30	0.70	0.99
g/kgBW^0.75^	102.37	101.84	99.88	94.39	2.68	0.30	0.65	0.93
Nutrient intake, kg/day								
Organic matter	5.83	5.80	5.78	5.39	1.42	0.31	0.53	0.76
Crude protein	0.85	0.85	0.85	0.79	0.02	0.32	0.55	0.76
Neutral detergent fiber	2.09	2.08	2.07	1.93	0.10	0.32	0.53	0.75
Acid detergent fiber	1.75	1.74	1.74	1.62	0.09	0.32	0.55	0.75
Nutrient digestibility, %								
Dry matter	78.12	77.31	79.38	79.36	1.23	0.61	0.87	0.66
Organic matter	77.50	77.51	79.36	79.39	1.22	0.63	0.85	0.65
Crude protein	87.43	86.22	89.54	87.37	2.07	0.86	0.91	0.59
Neutral detergent fiber	65.53	60.77	64.56	65.30	3.20	0.91	0.67	0.69
Acid detergent fiber	66.16	63.38	65.66	65.38	2.24	0.99	0.79	0.71

BSP = Brazilian spinach pellet; DM = Dry matter; SEM = standard error of the mean; Contrast = L, linear; Q, quadratic; C, cubic.

### 3.2 Rumen protozoa and blood urea nitrogen (BUN)

Brazilian spinach pellet supplementation has no appreciable impact on ruminal pH or NH_3_–N. Average rumen protozoal population linearly decreased (p = 0.002; Table 3) by BSP supplementation levels increased from 11.25 to 7.50 ( × 10^5^ cell/mL) at 0 h and 10.50 to 6.75 ( × 10^5^ cell/mL) at 4 h post-feeding, resulting in a decreased rumen protozoal population of 33 % at 0 h and 35.72 % at 4 h, with 8 % supplemented. For 8 % supplemented BSP in the substrate, the average protozoal population decreased by 34.47 %. Also, increasing the level of BSP from 12.50 to 13.25 mg/dL and then again from 12.75 to 13.29 mg/dL made the BUN rise by 5.66 % at 0 h before feeding and by 4.06 % at 4 h. This was a significant increase (p = 0.004; average BUN).

**Table 3:** Effect of Brazilian spinach pellet supplementation on rumen ecology in beef cattle.

Items	BSP supplementation (%) of DMI				SEM	Contrast		
	2	4	6	8		L	Q	C
Ruminal pH								
0 h pre-feeding	6.80	6.91	6.91	6.95	0.13	0.46	0.79	0.79
4 h post-feeding	6.74	6.97	6.92	7.01	0.14	0.23	0.63	0.51
Mean	6.77	6.94	6.91	6.98	0.11	0.25	0.65	0.57
Ruminal NH_3_–N, mg/dL								
0 h pre-feeding	19.26	22.42	23.82	24.17	2.37	0.15	0.56	0.95
4 h post-feeding	21.37	23.47	24.17	25.57	2.66	0.34	0.90	0.61
Mean	20.32	22.94	23.99	24.87	1.36	0.42	0.66	0. 91
Protozoa ( × 10^5^ cells/mL)								
0 h pre-feeding	11.25	9.50	8.00	7.50	0.47	0.003	0.21	0.73
4 h post-feeding	10.50	9.00	7.25	6.75	0.37	0.011	0.30	0.73
Mean	10.88	9.25	7.63	7.13	0.28	0.002	0.34	0.66
Blood urea nitrogen, mg/dL								
0 h pre-feeding	12.50	12.50	13.08	13.25	0.08	0.004	0.63	0.24
4 h post-feeding	12.75	13.00	13.50	13.69	0.06	0.003	0.81	0.35
Mean	12.63	12.75	13.29	13.47	0.06	0.004	0.80	0.15

BSP = Brazilian spinach pellet; DMI = Dry matter intake; SEM = standard error of the mean; Contrast = L, linear; Q, quadratic; C, cubic.

### 3.3 Ruminal VFA and CH_4_ estimation

Brazilian spinach pellet supplementation did not influence the concentration of total VFA, C2, C3, C4, or the C2:C3 ratio (p > 0.05; Table 4). The BSP supplementation had no influence on the CH_4_ estimate.

**Table 4:** Effect of Brazilian spinach pellet supplementation on volatile fatty acid in beef cattle.

Items	BSP supplementation (%) of DMI				SEM	Contrast		
	2	4	6	8		L	Q	C
Total volatile fatty acid, mmol/L								
0 h pre-feeding	104.48	107.85	105.01	111.96	1.29	0.12	0.50	0.19
4 h post-feeding	105.59	105.35	106.81	106.21	1.04	0.73	0.93	0.70
Mean	105.04	106.60	105.91	109.09	0.69	0.09	0.57	0.34
Acetic acid (C_2_), mmol/L								
0 h pre-feeding	62.51	62.14	61.39	61.74	0.60	0.8	0.77	0.78
4 h post-feeding	63.10	63.18	63.13	63.07	0.64	0.98	0.96	0.98
Mean	62.81	62.66	62.26	62.40	0.90	0.48	0.55	0.69
Propionic acid (C_3_), mmol/L								
0 h pre-feeding	21.60	22.36	22.66	22.83	0.44	0.33	0.74	0.93
4 h post-feeding	23.13	23.89	23.96	24.55	0.98	0.34	0.93	0.78
Mean	22.36	23.13	23.31	23.69	0.42	0.29	0.82	0.84
Butyric acid (C_4_), mmol/L								
0 h pre-feeding	15.89	15.50	15.95	15.43	0.62	0.87	0.96	0.75
4 h post-feeding	13.77	12.92	12.90	12.38	0.57	0.21	0.82	0.68
Mean	14.83	14.21	14.43	13.91	0.83	0.51	0.95	0.68
Acetic acid to propionic acid ratio (C_2_:C_3_)								
0 h pre-feeding	2.96	2.83	2.76	2.79	0.16	0.45	0.63	0.95
4 h post-feeding	2.70	2.62	2.62	2.54	0.14	0.46	0.99	0.80
Mean	2.82	2.73	2.69	2.67	0.14	0.43	0.77	0.94
Methane estimation, mmol/L								
0 h pre-feeding	29.09	28.38	28.04	28.25	0.97	0.52	0.64	0.96
4 h post-feeding	27.13	26.68	26.62	26.02	0.72	0.31	0.92	0.78
Mean	28.11	27.53	27.33	27.14	0.74	0.36	0.80	0.92

BSP = Brazilian spinach pellet; DMI = Dry matter intake; SEM = standard error of the mean; Methane estimation = (0.45 × acetic acid) - (0.275 × propionic acid) + (0.40 × butyric acid); Contrast = L, linear; Q, quadratic; C, cubic.

### 3.4 Nitrogen balance

The effects of BSP supplementation on N intake, N excretion, feces N excretion, urinary N excretion, N absorption, and N retention were not different between treatments (p > 0.05; Table 5).

**Table 5:** Effect of Brazilian spinach pellet (BSP) supplementation on nitrogen balance in beef cattle.

Items	BSP supplementation (%) of DMI				SEM	Contrast		
	2	4	6	8		L	Q	C
N intake, g/day	147.35	146.56	146.23	136.21	3.57	0.31	0.53	0.76
N excretion, g/day	43.53	45.22	47.22	44.03	1.90	0.84	0.53	0.75
Feces N excretion, g/day	20.48	24.32	23.29	20.82	1.41	0.99	0.29	0.79
Urinary N excretion, g/day	23.05	20.90	23.93	23.21	1.53	0.80	0.82	0.53
N absorption, g/day	126.87	122.24	122.93	115.40	3.21	0.26	0.83	0.65
N retention, g/day	103.82	101.34	98.99	92.18	3.27	0.22	0.74	0.88

BSP = Brazilian spinach pellet; DMI = Dry matter intake; SEM = standard error of the mean; Contrast = L, linear; Q, quadratic; C, cubic.

## 4 Discussion

The inclusion of the BSP in the current trial did not have any negative impact on the palatability of the diet. This observation was related to that revealed by Prommachart et al. [17], who discovered that the black rice and purple corn extracted residue (BPER) consists both phenolic acids and anthocyanin. The results showed that BPER supplementation had no effect on DMI, DM, OM, NDF, or ADF digestibility. Consequently, it is possible that the content of anthocyanin in treatment meals is not sufficient to interfere with the microorganisms that are present in the rumen [17]. Additionally, dairy cows that are milking did not have any changes to their total digestibility of nutrients or DMI when given anthocyanin corn silage [18]. It is possible that the data that have been reported suggest that anthocyanin does not have a detrimental impact on the amount of feed that ruminants receive. Anthocyanin is one type of flavonoid. Although it has a positive charge at the O_2_ atom of the C-ring, which is the structure of a flavonoid [19].

Under this investigation, this ruminal pH was in a good range of values to support rumen microbial activity. Flavonoids are recognized for their beneficial effects during periods of animal stress, maintaining a stable rumen pH in cases of subacute acidosis, and reducing inflammation caused by high grain diets. Additionally, their antibacterial properties are determined by their chemical structure, specifically the substitutions on the aromatic rings [20]. According to the findings, the pH level of the rumen ranged from 6.7 to 6.9, which is within the normal range. This is significant since the ideal pH range is between 6.5 and 7.0 [21]. A comparable finding was discovered by Sommai et al. [8], who discovered that the addition of flavonoid extract did not have any effect on the concentration of ruminal pH or NH_3_–N. Furthermore, the ruminal pH and NH_3_-N concentration of supplemental flavonoids derived from mulberry leaf flavonoids in sheep did not exhibit any significant changes [22]. This indicates that the quantities of flavonoid compounds that are supplemented do not have an effect on the pH of the rumen or the concentration of NH_3_–N.

The reduction in the population of protozoa that occurred when BSP was supplemented might be attributed to flavonoids' properties. Because of flavonoids diverse biological functions, including antibacterial capabilities, rumen pH regulation, modulation of protozoa, and reduction of methane emissions, they have garnered interest [7]. Nutritional strategies that aim to decrease the population have been beneficial to ruminants. Flavonoids have antibacterial properties and have an action on ruminal microbial populations [23]. Flavonoids work against microbes by impairing the action of the cytoplasmic membrane, inhibiting the microbial cell wall, or inhibiting nucleic acid synthesis. It was found by Ma et al. [22] that the ruminal population of protozoans and methanogens in sheep was reduced when the sheep were given a dietary supplement that contained flavonoids derived from mulberry leaf. *In vitro* research conducted by Oskoueian et al. [7] showed that flavonoids, specifically naringin and quercetin, had the effect of reducing the number of hydrogenotrophic methanogens and ciliate protozoa. When the amount of flavonoid extract provided increased from 0 to 40 mg, the protozoal population dropped in a linear fashion, as stated by Sommai et al. [8].

When the cattle were given BSP, the quantities of BUN in their blood increased, as demonstrated by this experiment. The BUN level was connected to what Totakul et al. [24] found when they added chaya leaf pellets to the diets as a source of tannins and saw that the BUN level ranged from 13.7 mg/dL to 14.3 mg/dL. Using fodder tree leaves as the source of flavonoid supplementation led to BUN values that ranged from 10.5 to 15.5 mg/dL, according to the findings of Bhatta et al. [25]. The optimal range for the average BUN content was between 10.0 and 15.0 mg/dL [26]. Blood urea-nitrogen concentration increased in correlation with NH_3_–N concentration, and diet treatments had an impact on BUN concentration, which ranged from 12.63 to 13.47 mg/dL. Foiklang et al. [27] mention that monesin could lower the NH_3_–N concentration in the rumen by slowing down the breakdown of peptides and amino acids. This is because monesin stops bacteria from growing that make too much ammonia. On the other hand, Krause and Russell [28] looked into the possibility that some of the gram-positive bacteria may be resistant to monensin. It was revealed that monensin did not have any effect on *Clostridium aminphilum,* which is a gram-positive bacteria that plays a role in the process of breaking down amino acids. When it comes to their activity, flavonoids, which are polyphenolic chemicals, are comparable to monensin and other forms of antibiotics [6].

The addition of the BSP level on top did not alter the overall VFA concentration, and the VFA profiles remained unchanged in the current investigation. Supplementation with flavonoid extract had no effect on C4 concentration, as shown by Sommai et al. [8]. Additionally, according to Cherdthong et al. [29], supplementing with *Piper sarmentosum* leaf powder did not affect C2, C4, or the C2:C3 ratio. Ampapon et al. [30], who used phytonutrient pellets in beef cattle, found that total VFA, C2, and C4 were similar among treatments. In addition, the estimate of CH_4_ was not affected by supplementation in this study under investigation. Molina-Botero et al. [31] found that putting dairy cows in respiration chambers with rutin (glucrohamnoside of quercetin) did not change how they used energy or reduced CH_4_. A wide variety of outcomes may be achieved by the utilization of secondary compounds for the reduction of CH_4_ production. These outcomes are contingent upon the group of compounds, the features of the compounds, and the amounts of the compounds. The fact that the amount of CH_4_ estimated did not change when BSP was added may be because BSP is not very concentrated, so it does not affect the methanogenic bacteria or the VFA in the rumen. Additionally, it is necessary to validate their impact on CH_4_ emission by long-term in vivo tests. This is because there is a chance that rumen microorganisms will adjust to the metabolite [20]. Moreover, in the rumen, C3 concentration was linked with H_2_ absorption, whereas C2 and C4 formation processes liberated H_2_ [32]. Thus, converting rumen fermentation from C2 to C3 will result in less H_2_ release and a decrease in CH_4_ emission [33]. This study discovered that BSP supplementation had no effect on the proportion of total VFA, C2, C3, C4, and C2:C3, which prevented dietary treatment from changing CH_4_.

N-balance was not significantly affected by increasing the level of BSP supplementation. In ruminants, N retention is used as a marker for the presence of proteins. The combined effects of NH_3_ absorption from the rumen and the recycling of that N back into the gut determined the urinary N output. In the rumen, both processes were correlated to the feed's constitution and the effectiveness of dietary N balance [34]. In the current investigation, there were no variations in the dietary elements or N balance. The high flavonoid content of dried kratom leaves (DKTL) did not have any effect on the total N output, the fecal N output, or the urine N output, according to the findings of Chanjula et al. [35]. It's possible that this is because DKTL supplementation slows down the pace at which N is broken down in the rumen and delays the excretion of N. Flavonoids derived from mulberry leaf and resveratrol were investigated by Chen et al. [3] for their effects on sheep. Resveratrol administration was found to have no effect on N retention while dramatically reducing fecal nitrogen, increasing nitrogen digestibility, and significantly increasing urine nitrogen excretion. However, supplementation with mulberry leaf had no discernible impact on N metabolism in sheep. It is shown that in this study, BSP did not affect N balance. The results found with the use of flavonoids for N balance are variable and dependent on the group of flavonoids and the dose of flavonoids.

## 5 Conclusion

The results of this research indicate that the addition of Brazilian spinach pellet did not have any discernible effect on the amount of feed utilized, the characteristics of the rumen, or the degree to which nitrogen was utilized. Notably, at supplementation levels of 6–8% of DMI, a reduction in protozoal population was observed, although methane estimation did not decline. Consequently, future research should explore higher supplementation levels (>8 %) of Brazilian spinach pellet in ruminant diets to thoroughly investigate its potential for methane reduction.

## Ethics statement

The authors attest that the journal's ethical rules, as stated on the author guidelines page, have been followed and that the required ethical review committee permission has been obtained. The authors affirm that they followed EU guidelines for the protection of animals used in research.

## Funding statement

The authors express their most sincere gratitude to the Research Program on Toxic Substances, Microorganisms and Feed Additives in Livestock and Aquatic Animals for Food Safety, Research Program on Research and Development of Winged Bean Root Utilization as Ruminant Feed, Increase Production Efficiency and Meat Quality of Native Beef and Buffalo Research Group, and Research and Graduate Studies, Khon Kaen University (KKU). Special thanks to Thailand Research Fund (TRF) through the 10.13039/501100017170Thailand Science Research and Innovation (TSRI), Thailand (TRF-IRN5702PHDW06).

## Data availability statement

Data will be made available on request.

## CRediT authorship contribution statement

**Sukruthai Sommai:** Writing – review & editing, Writing – original draft, Formal analysis, Data curation, Conceptualization. **Metha Wanapat:** Writing – review & editing, Project administration, Funding acquisition, Data curation. **Chanon Suntara:** Writing – review & editing, Writing – original draft, Formal analysis, Data curation, Conceptualization. **Rittikeard Prachumchai:** Writing – original draft, Formal analysis, Data curation, Conceptualization. **Anusorn Cherdthong:** Writing – review & editing, Validation, Supervision, Project administration, Funding acquisition, Conceptualization.

## Declaration of competing interest

The authors declare that they have no known competing financial interests or personal relationships that could have appeared to influence the work reported in this paper.
